# Hypothyroidism and mood disorders: integrating novel insights from brain imaging techniques

**DOI:** 10.1186/1756-6614-4-S1-S3

**Published:** 2011-08-03

**Authors:** Maximilian Pilhatsch, Michael Marxen, Christine Winter, Michael N Smolka, Michael Bauer

**Affiliations:** 1 Department of Psychiatry and Psychotherapy, University Hospital Carl Gustav Carus, Technische Universität Dresden, Dresden, Germany

## Abstract

**Abstract:**

Thyroid hormones play a critical role in brain development but also in the adult human brain by modulating metabolic activity. Hypothyroid states are associated with both functional and structural brain alterations also seen in patients with major depression. Recent animal experimental and preclinical data indicate subtle changes in myelination, microvascular density, local neurogenesis, and functional networks. The translational validity of such studies is obviously limited. Clinical evidence for neurobiological correlates of different stages and severities of hypothyroidism and effects of pharmacological intervention is lacking but may be achieved using advanced imaging techniques, e.g. functional and quantitative MRI techniques applied to patients with hypothyroidism before and after hormone replacement therapy.

## Introduction

The profound influence of thyroid hormones on brain development in humans has been studied extensively [1-6]. Hypothyroidism is a clinical condition which is either characterized only by elevated levels of the thyroid stimulating hormone (TSH; “subclinical hypothyroidism”) or additional low levels of trijodothyronine (T3) and thyroxin (T4) respectively (overt hypothyroidism). Taken together overt (0.4%) and subclinical (9%) hypothyroisism is prevalent in about 9.4 % of the adult population [7] and commonly affects brain function. Conversely, 15% of patients with depression display hypothyroid states including subclinical hypothyroidism [8], and about 25-30% of depressed patients show a pathological response to the thyreotropine releasing hormone (TRH) stimulation test [9].

The concepts of thyroid hormone action in the adult brain have changed dramatically along with rapid advances in basic science and methodological techniques over the past decades [10]. Particularly brain imaging techniques evaluating cerebral metabolism, perfusion, and anatomy enabled encouraging insights into the thyroid-brain relationship. As a result, it is now widely accepted that thyroid hormones play a critical role in the adult brain impacting mood and cognition. Even if crucial details of the underlying mechanisms still remain unknown [11], the therapeutic use of thyroid hormones in the clinical treatment of mood disorders has already been established in predominantly open trials with sometimes limited numbers of patients.

Thyroid hormone treatment has been shown to accelerate and augment antidepressant pharmacotherapy and to support maintenance therapy of bipolar affective disorders, even in patients with euthyroid hormonal state [10,12]. Specifically, there is good evidence that trijodthyronine (T_3_) can accelerate [13] and augment [14] the therapeutic response to tricyclic antidepressants and selective serotonin reuptake inhibitors (SSRIs) in depression.

Adjunctive treatment with supraphysiological doses of Levothyroxine (T_4_) was found to be effective in open clinical studies of patients with affective disorders [15-18]. In a subset of 21 patients with treatment-resistant affective disorder, very high doses of up to 600 μg per day L-T_4_ were required to achieve therapeutic response with a good tolerability even over mean treatment periods of up to 51 month [16].

In the first double-blind randomized placebo-controlled trial (RCT) to evaluate efficacy and tolerability of supraphysiological doses of L-T4 in 63 patients, we found that bipolar depressed women treated with L-T4 showed a better improvement in depression scores than those treated with placebo. However including both genders, the study failed to detect an overall statistically significant treatment effect (Stamm et al., in preparation).

## Brain imaging methods

Principal brain imaging techniques mentioned in this article are single photon emission computed tomography (SPECT), positron emission tomography (PET), and different methods of magnetic resonance imaging (MRI). PET and SPECT are nuclear imaging methods that require the injection of radioactive tracers into the circulatory system. Those tracers bind to molecular structures like transporters and receptors in the human brain. Measuring of the local distribution of the bound tracers allows an estimation of regional brain activation or availability of receptors. The glucose analog [^18^F]-Fluor-2-Desoxy-D-Glucose (FDG) is by far the most commonly used PET tracer and its metabolism reflects the amount of brain activity in different brain regions.

In contrast to these nuclear imaging methods, MRI is non-invasive and uses magnetic fields and radio waves instead of ionizing radiation. Structural MRI methods allow creating images of anatomical structures in an excellent spatial resolution (less than 1 mm). Diffusion tensor imaging (DTI) characterizes the mobility of water molecules and, thus, the directionality and integrity of white matter tracts. Magnetization transfer (MT) is sensitive to myelin content and is therefore useful in detecting early demyelination processes.

Functional magnetic resonance imaging (fMRI) has become the tool of choice to study functional aspects of the human brain. This method detects local increases of blood flow and the following decrease of deoxygenated hemoglobin during task related brain activity. The signal measured directly relies on the so-called BOLD (blood oxygen level dependent) effect. It is based on the different magnetic properties of oxygenated and deoxygenated hemoglobin. The origin of the BOLD signal is still a matter of discussion as it is also based on the complex interaction of neuronal metabolism, neuronal activity, blood flow and blood volume. Nevertheless fMRI enables the monitoring of active neuronal networks during the performance of specific paradigms, e.g. a memory task. Related to affective disorders, MRI allows the investigation of functional and structural deficits associated with neuropsychiatric symptoms as well as treatment effects.

Major advantages of neuroimaging methods are that brain structure and function as well as molecular and neurochemical processes can be studied in the living human. An important limitation of the mentioned brain imaging techniques refers to temporal resolution. While neuronal activities occur in the range of milliseconds, processes detected by imaging methods currently ranges between seconds (fMRI) and minutes (PET). Additionally, all methods measure brain activity indirectly through changes in blood flow or glucose uptake. Consequently, alterations in baseline perfusion and metabolic rate of oxygen may affect the results. Moreover it is currently not possible to reliably distinguish between inhibitory and excitatory neuronal activities. Nevertheless, given the rapid developments in neuroimaging methods; many limitations may hopefully be overcome in the next decades [19].

## Mechanisms of action

On the molecular level, thyroid hormones act via genomic and non-genomic effects. As part of the nuclear superfamily of ligand-modulated transcription factors, thyroid hormones bind to intracellular nuclear receptors [20]. Genomic actions of T_3_ are mediated through the control and usually increase of gene expression [12,21,22]. Genes that are regulated by thyroid hormones are known to encode for proteins such as myelin, neurotrophins, and proteins that are involved in intracellular signaling pathways [23].

Non-genomic actions of thyroid hormones have been described in the brain and peripheral tissues. After binding to cytoplasmic thyroid hormone receptors, T_3_ appears to be able to rapidly activate the phosphatidylinositol-3-kinase protein pathway and thereby achieves vasodilatory and neuroprotective effects [24]. Additionally, an increase in serotonergic neurotransmission is mediated by a thyroid hormone induced reduction of the sensitivity of 5-HT1A autoreceptors in the raphe nuclei and increase in 5-HT2 receptor sensitivity [25,26]. The exact role of these non-genomic signaling mechanisms, their regulation, and interaction with the slower genomic pathways remains to be elucidated.

Thyroid hormone homeostasis in the brain underlies a complex interaction of different autoregulative mechanisms [27,28]. The activity of specific thyroid hormone transporters (MCT8, LAT2) and the carrier transthyretin determines intracellular concentration of thyroid hormones via mediating their cellular influx and efflux under consideration of overall circulating levels of T_4_ and T_3_[27]_._ The activity of deiodinases [29,30] controls local bioavailability of T3 in concert with other less understood mechanisms, e.g. the local distribution of the different nuclear thyroid hormone receptors (TRα and TRβ in diverse isoforms) [31]. Thyroid hormone receptors are widely distributed in the brain with highest concentrations in cerebral cortex, hippocampus, amygdala, plexus choroideus and olfactory bulb [31].

Interestingly, the limbic structures, where thyroid hormone receptors are prevalent, have repeatedly been shown to be implicated in the pathogenesis of mood disorders [32]. However, the neuropharmacological basis and the functional pathways for the modulatory effects of thyroid hormones on mood are yet to be understood, even though several studies revealed interactions with different neurotransmitter systems, which are generally believed to play a major role in the regulation of mood and behaviour, i.e norepinephrine or serotonine [26,33].

## Structural changes induced by hypothyroidism

Thyroid hormones are known to affect neuronal differentiation, migration, myelination, synaptogenesis, and dendritic branching [8,9,34,35]. Therefore, hypothyroidism will change brain structure and the changes will depend on the onset time of the condition. Hypothyroid rodents display a loss of neurons in several brain regions. Next to the visual cortex [36] and cerebellum [37], the hippocampal formations were studied repeatedly. The number of granule cells in the dentate gyrus was reduced [38] with a more severe effect of hypothyroidism during development than during adulthood [39]. Rats treated with methimazole, a hypothyroidism inducing drug, showed a reduced count of pyramidal neurons in the CA3 hippocampal region [40]. This was found even if methimazole was administered in adulthood. Animals could be fully protected with T4 replacement and partially protected with T3 replacement [41]. It is speculated that this protective potency is mediated through the activation of hippocampal progenitor cells of the supraventricular zone and the dentate gyrus [42]. Mice with a heterozygous mutation of the thyroid hormone receptor alpha 1 showed a reduced density of GABAergic inhibitory interneurons in the CA1 hippocampal region [40]. On the behavioural side, these mice show increases in markers for depression and anxiety [43,44]. Both the behavioural measures as well as the neuronal density normalized with high levels of T3 administered in adulthood. In contrast to the above findings, hypothyroid rats treated at age 0-30 days with propylthiouracil (PTU) showed a loss of pyramidal neurons in CA1, which did not ameliorate after withdrawal of the drug and approximate normalization of serum T4 levels [45]. A thyroidectomy at day 30 also reduced the number of neurons. The same study reported in CA3 a volume decrease of the pyramidal layer combined with an increase in neuronal density and no loss in the total number of pyramidal neurons. Only one study is known in this context that applied MRI in rodents [46]. Using manual segmentation, perinatal hypothyroidism induced with methimazole administered to mother rats was found to reduced total brain size of the litter but not the relative size of the hippocampus.

Effects of T3 and T4 have also been reported in the process of myelination or remyelination in mice [47,48] and rats [49,50]. Harsan et al. [48] used Diffusion Tensor Imaging (DTI) in combination with histology to serve as a marker for remyelination in mice and found that cuprizone induced demyelination in adulthood was reversable through treatment with T3, which increased the number of oligodendrocyte progenitor cells. Using histology and electronmicroscopy, Franklin and Gilson [51] found that Schwann cell remyelination was decreased in thyroidectomized rats but not oligodendrocyte remyelination.

While the efficacy of thyroid hormone treatments is generally accepted, human data on structural changes in the brain is sparse. Using proton MRS, Jagannathan et al. [52] found that abnormalities related to myelin maturation were reversible under T4 therapy in three mentally disabled patients with congential hypothyroidism. A more recent investigation [53] using similar MRS and MRI methodology demonstrated general atrophy and signs of demyelination in two children with monocarboxylate transporter 8 (MCT8) gene deficiency, which is associated with a syndrome combining thyroid and severe neurological abnormalities.

Systematic group studies of structural brain changes due to hypothyroidism and thyroid hormone therapy are needed both during development and in adulthood. In addition to MRS, recent advances in structural MRI, quantitative MRI, and image analysis techniques could be utilized to test hypotheses derived from animal studies in human. Structural changes related to myelin can now be studied with quantitative T2 or quantitative magnetization transfer (MT) imaging [54]. Diffusion tensor imaging (DTI) can reveal information on white matter integrity. Changes in grey matter density [55] or cortical thickness [56,57] can be studied with T1-weighted images.

## Vascular Changes and Perfusion

Thyroid hormones are known to affect the vascular system. Hypothyroidism is associated with impaired fibrinolysis and blood coagulation resulting in cerebrovascular diseases [58]. It also compromises protective endothelial [59] and thrombocyte functions as well as lipid metabolism [60].

In the rat forebrain, a significant reduction in blood vessel density was observed in untreated rats thyroidectomised shortly after birth compared to euthyroid control animals and thyroidectomized rats treated with T4 or the thyroid hormone analog 3,5-diiothyroprionic acid (DITPA) [61]. Treatment induced angiogenesis and improved functioning of endothelial cells with secondary impact on local oxygen consumption and metabolic activity.

PET and SPECT measurements of cerebral blood flow under consideration of the thyroid system revealed partially conflicting results. Most consistently hypothyroidism was associated with global, diffuse hypoperfusion [62,63]. Several studies pointed to more regional effects including perfusion deficits pronounced in posterior brain regions [64-66] or in the parietal lobe [63].

Interpretation of the findings regarding reversibility of perfusion abnormalities under treatment is especially challenging. Whereas several studies report partial normalization of perfusion [17,62,67], others demonstrate [64-66] ongoing regional hypoperfusion under hormone replacement therapy. In contrast to the hypoperfusion pattern reported for hypothyroidism by Kraus et al. [64] in otherwise healthy subjects with posterior hypoperfusion and slight hyperperfusion anteriorly, depressed patients showed hypoperfusion in limbic, subcortical and frontal brain regions [32], which normalized under therapy with supraphysiological dosages (300 mcg/day) of T_4_.

Differences in the studied populations may be the reason for such variable findings. Published results refer either to severe cases of hypothyroidism [62,63] or mild cases [66,68]. Some studies excluded patients with co-morbid psychiatric diseases like depression [64,68], while other studies included also depressed patients [17,69]. In addition, the duration of the disease was variable. Only one study examined populations with varying severity and duration of the disease [17]. Taken together the existing data may indicate that long and severe hypothyroidism, presumably leading to irreversible structural neuronal and vascular changes, is associated with chronic perfusion and functional alterations, which may be ameliorated by treatment but not fully restored. In contrast, shorter and milder courses of hypothyroidism, presumably not being paralleled by irreversible structural changes, seem to be more accessible to substitution treatment.

## Glucose metabolism

In line with findings that general glucose metabolism is tightly coupled to perfusion [3], preclinical studies generally reported alterations of glucose metabolism in hypothyroid conditions [63,69]. More specifically, Bauer et al. related depressive symptoms in hypothyroidism to regionally decreased glucose metabolism, particularly in the peri-genual anterior cingulate cortex region and the hippocampus [17] in a glucose-PET study. After 3 months of T4 substitution therapy, activity patterns normalized. Circulating TSH levels before initiation of therapy showed a significant positive correlation with glucose metabolism in the right ACC and negative correlations in the hippocampus. Interestingly, these two regions express TSH receptors very densely according to topographic studies in rat and human [70], whereby suggesting a direct and relevant effect of elevated TSH levels on the limbic system is of course highly speculative.

## Cognitive and emotional functional changes

In line with the wide range of metabolic, perfusional and structural implications of the thyroid system, the literature on functional alterations related to thyroid diseases is large. Disturbances of the thyroid system, particularly if leading to a hypothyroid state, may profoundly alter mental functions influencing cognition and emotions. Severe hypothyroidism may lead to both severe depression and dementia [71] especially if left untreated [72].

Neuropsychologically, several cognitive defects in general intelligence, psychomotor speed, visual-spatial skills, working and long-term memory have been observed ranging from minimal to severe [73-75]. It was suggested that hypothyroid-related memory defects are not attributable to an attention deficit but rather to specific retrieval deficits [73,76]. Motor skills, language, inhibitory efficiency, and sustained attention appear to be less impacted by hypothyroidism [73,75]. Older adults were more vulnerable to cognitive changes due to hypothyroidism [75].

It is still a matter of discussion as to whether subclinical hypothyroidism is associated with cognitive impairments. In some studies, patients with subclinical hypothyroidism performed worse than controls on well established neuropsychological tests such as the Wechsler Adult Intelligence Scale or the Wechsler Memory scale [77,78], while other studies found no association between subclinical hypothyroidism and several aspects of cognition [75,79,80]. A consistent finding among many studies was a specific deficit in working memory tasks [73]. These neuropsychological findings were additionally underlined by a functional MRI (fMRI) study by Zhu et al. [68] who found that working memory was impaired in patients with subclinical hypothyroidism but not other memory functions. These impairments were reversible with l-thyroxine (L-T_4_) treatment.

Despite clinical evidence of an increased vulnerability for affective disorders coming along with hypothyroid states, neurobiological correlates of this disposition are not systematically investigated yet. This is of particular interest because a study by Maheu et al. [81] demonstrated that fMRI could detect greater hippocampal and amygdala activation in 12 non-depressed adolescent patients with Cushing's syndrome, when encoding affective stimuli (i.e. emotional faces) compared to 22 healthy controls. A similar pattern of brain activation has previously been demonstrated in adolescents suffering from manifest depression in an equivalent paradigm [82]. CushingÂ´s syndrome is defined by hypercortisolemia and highly associated with prevalence of depressive symptoms in adults [83] and shows in this respect distinct parallels to hypothyroidism.

## Conclusions

Hypothyroid states have a multitude of effects on structure, perfusion and function of the central nervous system. Recent research aims to combine modern brain imaging techniques with years of experience in neuropsychological and clinical evaluations of thyroid dysfunctions. The results are encouraging, and the description of the interactions between thyroid and affective disorders is taking on a new dimension. Nonetheless, the potential of modern techniques has hardly been exhausted. For example, non invasive measurements of brain perfusion like arterial-spin-labeling (ASL) – already established in characterisation of different cerebrovascular diseases [84] – would be suitable for transferring hypotheses about the influence of angiogenesis [85] from animal models into humans. fMRI in patients with thyroid diseases of different length and severity could help to identify functional aberrations such as memory impairments or altered emotional processing, which has long been suggested from animal studies. Structural changes related to myelin, which have been observed in various animal models, can now be studied with quantitative T2 or quantitative magnetization transfer (MT) imaging [86]. Finally diffusion tensor imaging (DTI) [54] can reveal information on white matter integrity.

## Competing interests

The authors declare that they have no competing interests.

## Authors' contributions

MP participated in the design and the preparation of the manuscript. MM participated in the preparation and revision of the manuscript. MNS, CW and MB participated in its design, revisions and coordination. All authors read and approved the final manuscript.
